# Assistive Technologies for Communication Empower Patients With ALS to Generate and Self-Report Health Data

**DOI:** 10.3389/fneur.2022.867567

**Published:** 2022-04-26

**Authors:** Ana Londral

**Affiliations:** ^1^Value for Health CoLAB, Lisbon, Portugal; ^2^Comprehensive Health Research Center, Nova Medical School, Nova University of Lisbon, Lisbon, Portugal

**Keywords:** assistive technologies, amyotrophic lateral sclerosis, neurodegenerative conditions, patient-generated health data, communication, biomarkers

## Introduction

Amyotrophic Lateral Sclerosis (ALS) is a progressive neuromuscular disease with rapid and generalized degeneration of motor neurons. Patients with ALS experience a relentless decline in functions that affect the performance of most activities of daily living, such as speaking, eating, dressing, walking, and writing (1). The management of the respiratory system is the main concern of medical support, since respiratory failure is the most common cause of death in patients with ALS (2). In severe stages, as strategies to maximize the survival of patients with ALS are taken, the total locked-in syndrome may occur (3). Although there is growing evidence that mild cognitive impairment is common (44), most patients are self-conscious of their limitations. For this reason, this *disease of losses* (4), raises a deep concern amongst caregivers and patients themselves in preserving autonomy, self-control, and decision-making possibility for as long as possible. Assistive technologies (ATs) can support patients in preserving autonomy and control along with the disease progression.

Assistive technologies are of great impact to ALS patients, since their use may help to overcome severe functional limitations (5). There are many technology options available to support persons with neurodegenerative conditions, either mainstream or specifically designed products. In this study, we focus on ATs that specifically support patients in communicating, also denominated technologies for Augmentative and Alternative Communication (AAC). Indeed, everyday digital technologies of the last decade, such as smartphones, tablet devices, and the Internet, may be used to assist persons with neurodegenerative conditions in performing daily tasks, such as using voice-activated commands to control the environment and text-to-speech to communicate verbally.

As speech intelligibility declines (6), support in communication is important in ALS management, as communication using natural speech becomes difficult and frustrating (7). Caregivers experience increased frustration due to difficulties in understanding their partners' needs and increased dependency (8). Indeed, 80 to 95% of people with ALS are unable to meet their communication needs using natural speech, from a certain point of the disease progression (9). This means that, if no other resources than natural speech are used to communicate, patients will be deprived of expressing needs or feelings, making decisions, and keeping social relationships at some stage of the disease (10, 11). There is research evidence that the use of ATs to support communication has a positive impact on the quality of life of both patients and caregivers (12–14).

The field of ATs that support communication is well-developed for ALS patients. As patients start experiencing dysarthria (15), speech therapists and rehabilitation engineers support strategies and technologies to augment or replace speech communication (9). Due to the neurodegenerative characteristics of ALS, ATs need to be adapted as the patients' functional abilities decrease. Sensors to detect small movements or electrophysiological signals [e.g., brain-computer interfaces (BCI)], eye trackers, text-to-speech technologies, and software with screen keyboards and dynamic tables are among the main assistive communication technologies that are used to assist ALS patients (11). In the late stages, the use of the so-called *low-tech* assistive communication applications, such as a article letter board, is frequent (4).

This research presents a viewpoint on the importance of ATs in keeping ALS patients connected. ATs can empower patients to use telemedicine services to report outcomes and needs, during the full cycle of care, from diagnosis to death. When an ALS patient is enabled to use the internet, it will be possible to keep in contact with the patient, also in the late stages. In the author's perspective, this permanent possibility of contact includes an underexplored mean to achieve a better and more granular knowledge of the disease progression related to neurophysiology, symptoms, and patients' needs, therefore increasing patients' empowerment for data-reporting and decision-making, and also potentiates longitudinal patient-generated health data (16) that may be relevant to identify biomarkers related to the disease.

## Patients that use at are Empowered to Connect With Clinical Teams

**Figure 1:**
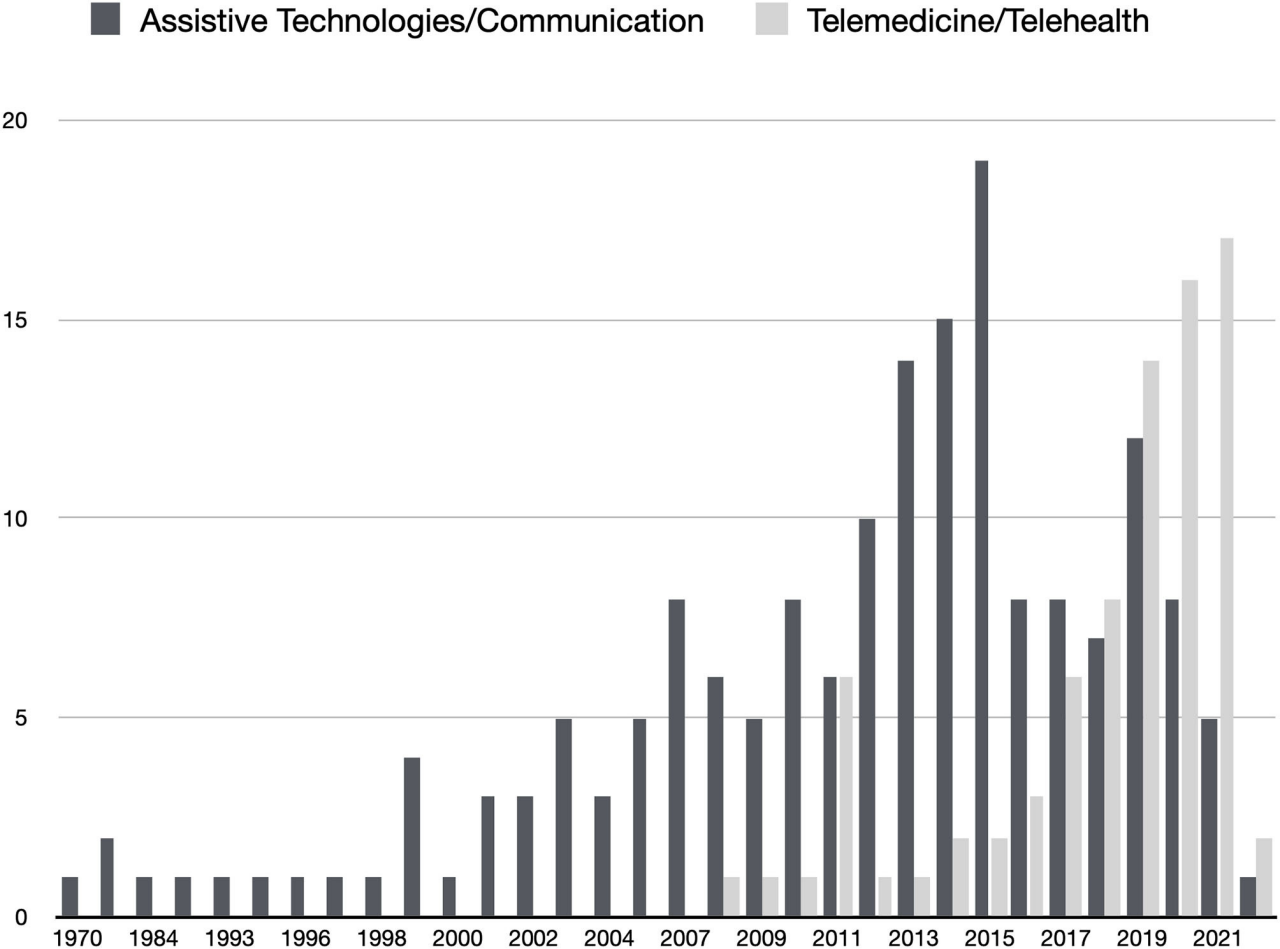
Results of two searches in Pubmed (light gray) ((telehealth) AND (amyotrophic lateral sclerosis)) OR ((telemedicine) AND (amyotrophic lateral sclerosis)); (dark gray) ((assistive technologies) AND (amyotrophic lateral sclerosis) AND (communication)). Date of search: 31/01/2022.

The COVID-19 pandemic disrupted healthcare systems in the general use of emergent telemedicine services (17). But before the SARS-CoV-2 crisis, telehealth services were already considered important for ALS patients. Studies reported the high adoption and adherence from patients and caregivers to telehealth for home monitoring and follow-up (18), but a more reserved attitude from healthcare professionals (19). Telehealth services, namely video calls, text messaging, self-reporting/self-monitoring, and remote non-invasive ventilation (NIV) monitoring increase safety, accessibility, and the quality of care. From a search in PubMed of articles containing the keywords “((telehealth) AND (amyotrophic lateral sclerosis)) OR ((telemedicine) AND (amyotrophic lateral sclerosis))” it is possible to observe that research publications increased from 2019, revealing increased interest in these services (Figure 1). Despite the high adherence of telemedicine in pandemic times, cost-effectiveness analysis is needed (19) to promote a sustained adoption from healthcare systems and teams.

As dysarthria aggravates and mobility limits patients to the home context, computers, and mobile devices are the instruments to communicate and stay connected to social and care networks (20). Limited mobility, assistive breathing, and feeding difficulties lead to enormous difficulties in going to clinical appointments as the disease progresses. In consequence, the accessibility of patients to a multidisciplinary care team decreases, and clinical experts may lose contact with patients in later stages. Communication difficulties between clinical teams and patients may also hinder retention in clinical trials (21), affecting research for better care and disease management.

When patients manage their own AT device, they can keep the communication with the healthcare professionals, allow a better assessment and monitorization of their symptoms and needs, then a better healthcare support. They are empowered to use telemedicine services for longer periods in disease progression, with benefits to their active participation in disease management, as also in clinical research.

A search in PubMed of articles containing the keywords “((assistive technologies) AND (amyotrophic lateral sclerosis) AND ((communication) OR (AAC))” demonstrates that, despite ATs and communication research exist for many years, a retraction is observed in the last years (Figure 1). This retraction in research may be related to the difficulties in funding ATs for ALS patients and in involving clinical teams in the empowerment of patients, often delegating communication and reporting to caregivers (8). Moreover, in the European context, after the enthusiasm for eye-tracking and BCI research for accessibility, there has been an absence of funding for research on these topics. It is worth to note the contradictory detachment between ATs and Telemedicine enthusiasm in research, observed in the last years, since it is important to understand that ATs enable patients to use telemedicine services.

## Patients that use at are Empowered to Self-Report and to Make Decisions

Several clinical instruments to monitor patients were already validated to be self-reported and remotely assessed, by phone or computer/smartphone (22–24). While some data can be passively collected from the patient (e.g., NIV parameters where data is generated and sent automatically), instruments for self-reporting need voluntary action from patients. For reporting, patients need access to a mobile device or a computer or a telephone to open a video call, or fill-in online questionnaires, or simply talk on the phone.

But, as disease symptoms progress, patients will have increasing difficulties in using a keyboard (either physical or touchscreen-based) and need to use input devices that do not rely on upper limb movements or speech (e.g., eye tracking or biosignals-based interfaces) (12, 25–28). These input devices are part of ATs for communication and allow patients to generate health data through computers or mobile devices. When patients are not able to use such devices, due to a lack of proper interfaces, they are disempowered to self-report symptoms and needs and increase their dependency on caregivers to make decisions (8).

Evaluation instruments that can be accessed and filled-in by patients through AT tools can support novel longitudinal research, and contribute to assess value in their health pathway. The support to patients for the use of alternative input devices empowers their active participation and collaboration in health data collection and decision-making during the full cycle of the disease.

## Patients that use at are Empowered to Generate Longitudinal Health Data

The identification of markers of disease progression is important to monitor ALS patients, with potential application in clinical trials (29, 30). ALSFRS-R is an assessment instrument that is widely used to mark disease progression, based on self or clinical reported symptoms' observation. Staging models capture disease progression (31). But these instruments do not provide continuous objective scoring and are not very sensitive to change, providing stages or relatively small slopes of decline (32, 33). For example, speech rate decreases prior to a perceived impact on speech intelligibility, but the first may be a marker for the latter, as studied by Ball et al. (34).

Previous research suggested mobile and computer devices as instruments to, objectively and with high sensitivity, capture disease progression in the daily life of patients. Signal processing of patients' speech recording from mobile devices allows a longitudinal identification of markers of dysarthria progression (7, 35, 36). The use of keys or buttons in physical or touchscreen devices can be used to capture the progression of ALS, mostly involving movements to select, tap or press and release keys/buttons (37). Eye tracking devices have been used to objectively assess extra motor cerebral involvement in ALS, by evaluating anti-saccade, trail-making, and visual search tasks (38). BCIs were used to assess cognitive function in patients with ALS who are severely disabled (39, 45, 46). A combination of eye-tracking and BCIs was proposed as a setup to apply a neuropsychological battery for cognitive assessment in ALS (40).

ATs for communication can be further explored as tools for in-home monitoring of disease progression. These devices can be used to support recording tools to continuously monitor speech, cognitive, and motor functions even prior to self-perceived symptoms. The possibility of monitoring the physiological, functional, and behavioral measures through patient-generated health data will help researchers to discover new biomarkers for disease progression. Ultimately, researchers are empowered when patients are empowered to report and generate data by themselves. This can be accomplished by the use of ATs.

## Discussion

Research on how people interact with technology and the increasing digital transformation of society are leading to a more comprehensive approach to the design of technologies that engage patients, their caregivers, and health professionals. While traditionally, technologies developed for healthcare were exclusively for the use of healthcare professionals or researchers, presently, technologies that are also used by the patients and improve the flow of information and communication between all parts (patients, caregivers, and healthcare teams) are providing novel data and experiences in healthcare. Patients are gradually going from passive recipients to active agents of their health (41). In fact, when ALS patients manage their own ATs device, they can preserve communication with the healthcare professionals along the full cycle of care and allow a better assessment and monitorization of their symptoms and needs. Patients are also empowered to participate in research studies that aim at identifying new biomarkers in their daily context and improving future care.

Resources and funding mechanisms for ATs differ in different countries. Complex and bureaucratic processes are a critical factor to access the support of AT, despite the increasing variety of solutions and information sources (20, 42). Due to poor funding, latency of provision, and lack of indication criteria in international ALS treatment guidelines, among other barriers (43), assistive technologies are difficult to access.

This opinion article emphasizes the relevance of providing assistive technologies to ALS patients beyond functional communication. From mainstream mobile devices to specific input devices based on electrophysiological sensors, ATs are tools that empower patients to actively generate health data that will support research for new clinical decision support tools toward assessment, monitoring, and care of neurodegenerative disorders. It is important to further research on technologies and strategies to support the communication and connection of ALS patients, merging novel perspectives and potential benefits of ATs as instruments for clinical research and high-value healthcare.

## Author Contributions

The author confirms being the sole contributor of this work and has approved it for publication.

## Funding

The author acknowledges funding from the Portuguese National Funding Agency for Science, Research, and Technology (FCT) and public ESF funding with references LISBOA-05-3559-FSE-000003 and DSAIPA/0106/2019/02.

## Conflict of Interest

The author declares that the research was conducted in the absence of any commercial or financial relationships that could be construed as a potential conflict of interest.

## Publisher's Note

All claims expressed in this article are solely those of the authors and do not necessarily represent those of their affiliated organizations, or those of the publisher, the editors and the reviewers. Any product that may be evaluated in this article, or claim that may be made by its manufacturer, is not guaranteed or endorsed by the publisher.
